# Antibiotics for Intra-Abdominal Infections: When, Which, How and How Long?

**DOI:** 10.3390/antibiotics14111127

**Published:** 2025-11-07

**Authors:** Massimo Sartelli, Miriam Palmieri, Francesco M. Labricciosa

**Affiliations:** 1 Department of General Surgery, Macerata Hospital, 62100 Macerata, Italy; miriam.palmieri@sanita.marche.it; 2 Global Alliance for Infections in Surgery, 62100 Macerata, Italy; labricciosafrancesco@gmail.com

**Keywords:** antibiotic therapy, antimicrobial resistance, intra-abdominal infections, surgery

## Abstract

Intra-abdominal infections (IAIs) remain among the most challenging problems in surgical clinical practice. They range from uncomplicated appendicitis to life-threatening peritonitis, demanding rapid diagnosis, timely source control, and appropriate antibiotic therapy. Antibiotics are crucial to manage patients with complicated IAIs. Antibiotics should always be prescribed appropriately, ensuring the correct spectrum, timing, duration, and dosage. Appropriate prescribing within hospitals enhances treatment success and patient safety, while also reducing the risk of opportunistic infections such as *Clostridioides difficile* and lowering the likelihood of selecting resistant pathogens. Over recent decades, antimicrobial resistance has escalated into a worldwide public health threat. The rapid rise in multidrug-resistant organisms, especially Gram-negative bacteria, has created a pressing global concern. The objective of this narrative review is to describe (a) *when* antibiotics should be used in patients with IAIs; (b) *which* antibiotics should be selected in patients with IAIs; (c) *how* they should be managed in patients with IAIs; and (d) *how long* they should be administered in patients with IAIs.

## 1. Introduction

Intra-abdominal infections (IAIs) remain among the most challenging problems in surgical clinical practice. They range from uncomplicated appendicitis to life-threatening peritonitis, demanding rapid diagnosis, timely surgical intervention, and judicious antimicrobial therapy. Antibiotics are indispensable in their management. For decades, the default response to IAIs has been broad-spectrum empiricism, often with prolonged courses of multiple agents. While this approach has undoubtedly saved lives, it has fostered collateral damage such as the selection of multidrug-resistant organisms and disruption of the microbiome [1].

The cornerstone of IAI treatment is source control [1]. No antibiotic can compensate for undrained abscesses or devitalized bowel. Once control is achieved, evidence shows that shorter antibiotic courses, sometimes as brief as four days, are as effective as traditional extended regimens. This paradigm shift underscores the importance of tailoring therapy to disease severity, host response, and microbiologic risk. Equally critical is the choice of agent. In community-acquired infections, narrow-spectrum coverage is often sufficient, avoiding unnecessary exposure to carbapenems or anti-pseudomonal agents. In contrast, healthcare-associated IAIs, particularly in patients with prior antibiotic exposure or critical illness, may necessitate a broader spectrum of antibiotics, followed by de-escalation once cultures clarify the microbiologic landscape [1]. Ultimately, antibiotics in IAIs demand a balance between urgency and precision, lifesaving therapy and the long-term threat of resistance. The challenge for clinicians is not simply to prescribe antibiotics, but to prescribe them wisely, as an act of stewardship as much as of care. Antibiotic therapy should begin once a treatable infection is identified or strongly suspected and should be personalized based on the patient’s clinical conditions and the risk of resistance patterns.

The objective of this narrative review is to describe (a) *when* antibiotics should be used in patients with IAIs; (b) *which* antibiotics should be selected in patients with IAIs; (c) *how* they should be managed in patients with IAIs; and (d) *how long* they should be administered in patients with IAIs.

## 2. When

IAIs are commonly classified into two categories: complicated and uncomplicated. The uncomplicated type typically involves only one organ and does not extend into the peritoneal cavity. When the infectious source is completely removed through surgery, further antibiotic therapy is not required, as demonstrated in cases such as uncomplicated acute appendicitis and cholecystitis [2,3,4].

Although appendectomy is considered the standard treatment for acute appendicitis, there has been a growing shift towards using antibiotics as an initial management strategy. Antibiotic therapy is regarded as a safe and effective option for patients with uncomplicated appendicitis without appendicolith. Nonetheless, its long-term success is limited due to significant recurrence rates and a potential risk of perforation, particularly when preoperative diagnosis via computed tomography (CT) is delayed or not performed accurately [5,6]. Consequently, non-surgical management should be reserved for selected patients, while surgery continues to represent the mainstay of treatment [7].

In cases of acute cholecystitis, surgery remains the preferred approach. Two main strategies exist for uncomplicated disease: an early laparoscopic cholecystectomy carried out within a few days of symptom onset during the same hospital admission, provided the diagnosis and patient’s surgical fitness are confirmed, and a delayed procedure performed after 6–12 weeks once acute inflammation has subsided [8]. The role of antibiotics in this context is less well defined than in appendicitis; however, their use tends to be short-term in early surgery and more prolonged in delayed surgery. A systematic review and meta-analysis by Lyu et al., which included 15 randomized controlled trials (RCTs) and 1669 patients, found that early laparoscopic cholecystectomy was equally safe and effective as delayed surgery for acute cholecystitis when performed within seven days of presentation [9]. No differences were observed in bile duct injury, wound infection, overall complications, or the need to convert to open surgery. However, early surgery was associated with a shorter hospital stay, though postoperative hospitalization was not significantly different [9]. A 2021 meta-analysis did not confirm that immediate cholecystectomy within 24 h of admission lowered complication rates [10]. Literature reviews further indicated that definitions of “early” surgery varied, ranging from within 24 h of admission to up to one week from symptom onset. Current evidence supports a seven-day window from presentation for performing early cholecystectomy [10]; however, a review published in 2022 concluded that in most patients, laparoscopic cholecystectomy, performed within 3 days of diagnosis, is the first-line therapy for acute cholecystitis [11].

The role of antibiotic therapy in uncomplicated acute diverticulitis has been the subject of considerable debate. In 2015, the World Society of Emergency Surgery (WSES) proposed a CT-based classification for acute diverticulitis of the left colon to aid clinical decision-making. This classification distinguishes uncomplicated diverticulitis, confined to the colon, from complicated disease, where the infection spreads beyond it [12]. Several studies have since demonstrated that in mild cases of uncomplicated diverticulitis, antibiotics provide no clear benefit over conservative treatment without antibiotics in terms of symptom resolution. The prevailing consensus is that, in immunocompetent individuals, uncomplicated diverticulitis is often self-limiting, with host defenses capable of resolving the inflammatory process without antibiotic support [13]. A multicenter randomized trial by Chabok et al. [14] in 2012, involving 623 patients, compared treatment with (314 patients) and without antibiotics (309 patients). The study concluded that antibiotics neither accelerated recovery nor prevented complications or recurrence in uncomplicated diverticulitis [14]. As a result, antibiotics are now recommended mainly for complicated cases. A prospective cohort study of 161 patients with CT-confirmed uncomplicated diverticulitis further demonstrated the safety of non-antibiotic management during a 30-day follow-up. Most patients (87%) were treated as outpatients, with only a small number requiring hospital admission. No progression to complicated diseases or surgical intervention occurred, though 14 patients (9%) eventually received antibiotics [15]. More recently, the Dutch DIABOLO randomized controlled trial, which compared observation against systemic antibiotics in patients with a first episode of CT-proven Hinchey 1a and 1b acute left-sided diverticulitis, confirmed that observational management did not delay recovery and was a safe alternative [16].

In cases of complicated intra-abdominal infections (cIAIs), the infection extends beyond the organ into the peritoneal cavity, resulting in either localized or diffuse bacterial peritonitis. Management requires both effective source control and appropriate antibiotic therapy. Antibiotics play a key role in preventing further local or systemic spread and in reducing late-stage complications. In patients with cIAIs, antibiotic treatment is usually initiated empirically, since conventional microbiological cultures require at least 24 h to identify pathogens and determine their susceptibility profiles. The patient’s clinical condition should guide the timing of therapy. In non-critically ill patients, empiric antibiotics should be started once an IAI is confirmed or strongly suspected. Evidence of a survival benefit from early empiric therapy remains inconsistent, even in cases of Gram-negative bacteremia [17]. By contrast, in patients with sepsis or septic shock, the rapid initiation of appropriate empiric antibiotics is crucial, as it has a major impact on clinical outcomes, independent of the infection site. According to the current literature, a strong correlation exists between each hour of delayed administration of appropriate antibiotic therapy and mortality in patients with septic shock. However, this relationship is less pronounced in patients with sepsis who do not experience shock [18,19].

Microbiological testing plays a pivotal role in determining therapeutic strategies and guiding targeted antibiotic therapy. Such testing enables clinicians to tailor antibiotic regimens—expanding coverage when initial treatment is too narrow, but more frequently de-escalating from unnecessarily broad empiric therapy. Although culture results have limited impact on the management of common community-acquired IAIs (CA-IAIs), such as acute appendicitis [20,21], the current context of widespread multidrug-resistant (MDR) organisms in both hospital and community settings makes antimicrobial resistance (AMR) a critical consideration. Microbiological testing is particularly valuable in hospital-acquired IAIs, where the risk of resistant pathogens is substantial. In critically ill patients, therapy should be reassessed based on culture and susceptibility findings. Expert recommendations suggest obtaining at least two sets of blood cultures before initiating antimicrobial treatment in hospitalized critically ill patients, as this approach improves diagnostic yield, with pathogens identified in more than half of tested cases [22,23]. This rate is markedly higher than the 6% reported by Montravers et al. [24]. Beyond guiding therapy in patients at risk for AMR, microbiological testing is also essential for improving knowledge of local epidemiology.

## 3. Which

In IAIs, choosing an inappropriate initial antibiotic regimen is linked to higher clinical failure, longer hospitalization, and increased costs compared with starting the right therapy from the outset [25,26,27,28]. Until the causative organism(s) and susceptibilities are known, empiric selection should be guided by local resistance patterns, patient-specific risk factors for resistance, and any available microbiological information (e.g., known colonization).

For CA-IAIs, pathogens usually reflect the patient’s endogenous flora and are relatively predictable. *Enterobacterales* (notably *Escherichia coli* and *Klebsiella* spp.), viridans group streptococci, and anaerobes—especially *Bacteroides fragilis*. *Enterococcus* spp.—are also frequently isolated in CA-IAIs, though their exact pathogenic contribution remains debated [1]. In an analysis by the Dutch Peritonitis Study Group of RELAP trial participants, the isolation of Gram-positive cocci—predominantly *Enterococcus* spp.—was associated with worse outcomes [29], even though microbiology did not reliably predict persistent abdominal infection after emergent laparotomy in cIAIs [30].

Key predictors of resistant organisms in cIAIs include healthcare-associated acquisition, recent antibiotic exposure, prior infection with MDR organisms, and intestinal colonization by resistant strains [31]. Post-operative peritonitis carries higher mortality due to severity, comorbidities, atypical presentations, and a greater likelihood of resistant pathogens [32].

AMR has intensified globally over the past two decades, with resistant Gram-negative infections driving morbidity and mortality. In postoperative peritonitis, Augustin et al. found, among 100 intensive care unit (ICU) patients, that administration of broad-spectrum antibiotics between the index procedure and reoperation was the only independent risk factor for the emergence of resistant bacteria [33]. In another retrospective ICU cohort of 242 cIAI patients (88 community-acquired; 154 post-operative), *Enterococci* were isolated most frequently (47.1%), followed by *E. coli* (42.6%), other *Enterobacterales* (33.1%), anaerobes (29.8%), and *Candida* spp. (28.9%). Susceptibility rates were lower in post-operative than community-acquired cases [34].

A five-year prospective ICU cohort examining secondary and tertiary abdominal sepsis characterized the peritoneal microbiology in detail [35]. Aerobic Gram-negative bacteria (AGNB) were present in 53% of peritoneal cultures, with *E. coli* comprising 45% of AGNB; 36% of patients harbored more than one AGNB. AGNB were most frequent in colorectal perforations (68.6%) and perforated appendicitis (77.8%), but least so in gastroduodenal perforations (20.5%). Gram-positive organisms were common in colorectal perforations (50%). *Candida* spp. were found in 19.9% (59.1% *C. albicans*), with higher rates in gastroduodenal (41%) and lower in colorectal perforations (11.8%). Anaerobes were cultured in 77.8% of perforated appendicitis. Montravers et al. showed that with each re-operation for persistent peritonitis, MDR carriage rose—from 41% at index surgery to 49%, 54% (*p* = 0.037), and 76% by the third re-operation (*p* = 0.003 vs. index)—supporting routine intraperitoneal sampling at every re-intervention [36].

In a multicenter, one-year prospective study, Maseda et al. stratified cases of healthcare-associated infections (HAIs), CA-IAIs, and immunocompromised hosts. Overall, 11.0% had AMR (7.0% extended-spectrum beta-lactamase [ESBL]/carbapenemase-producing *Enterobacterales* [CPE]), with substantially higher rates in HAIs (35.4%) vs. CA-IAIs (5.8%) and none in immunocompromised patients (0%). Thirty-day mortality was 14.5% overall—higher in HAI (23.1%) than CA-IAI (11.6%)—and was associated with age > 75 years, *Candida* isolation, and elevated SAPS II; biliary infections had lower mortality [37].

Across both community and hospital settings, ESBL-producing *Enterobacterales* represent the most prevalent resistance threat and are increasingly encountered in the community [38,39]. ESBLs hydrolyze many β-lactams, types of penicillin, aztreonam, and first- to third-generation cephalosporins, but not cephamycins or carbapenems [40,41]. Because ESBL genes are often plasmid-borne, co-resistance to aminoglycosides and fluoroquinolones is common [41]. Risk factors for ESBL infection include: (1) ≥48 h hospitalization within the prior 90 days; (2) ≥5 days of broad-spectrum antibiotics within 90 days; (3) ESBL gut colonization within 90 days; (4) exposure to healthcare settings with high MDR prevalence (e.g., long-term care) [42].

For severe ESBL-*Enterobacterales* infections, carbapenems remain the empiric mainstay. To curb carbapenem use, de-escalation to piperacillin–tazobactam may be considered when the MIC ≤ 8 mg/L (EUCAST), but evidence is mixed. In MERINO, 30-day mortality was higher with piperacillin–tazobactam versus meropenem for ceftriaxone-resistant *E. coli/K. pneumoniae* bloodstream infections (BSIs) (12.3% vs. 3.7%, *p* = 0.90) [43]; multiple methodological concerns have since been raised, including suboptimal PK/PD with 1 h infusions and E-test misclassification related to OXA-1 [44,45]. MERINO-2 (chromosomal AmpC producers) found more microbiologic failures with piperacillin–tazobactam than meropenem, though fewer relapses [46]. While several observational studies showed no mortality difference between piperacillin–tazobactam and carbapenems in ESBL BSIs [47,48,49,50], expert guidance typically favors a carbapenem (imipenem/meropenem) for severe third-generation cephalosporin-resistant infections in critically ill patients [51].

Tigecycline is a glycylcycline antibiotic that inhibits bacterial protein synthesis by binding to the 30S ribosomal subunit [52]. This mechanism allows tigecycline to evade traditional tetracycline resistance determinants, making it an interesting option for a therapeutic antibiotic for infections caused by multidrug-resistant (MDR) pathogens.

Tigecycline has attractive coverage for anaerobes, enterococci, and many ESBL producers, achieving high tissue distribution [52]. Yet meta-analyses of phase 3–4 trials observed higher all-cause mortality with tigecycline versus comparators in aggregate; subsequent modeling implicated host and infection-site factors rather than tigecycline itself [52]. Given its poor performance in bacteremia but high tissue distribution, tigecycline can be considered as a β-lactam-sparing component for IAIs, particularly in combination, when secondary bloodstream infection is a concern.

Aminoglycosides are potent against aerobic Gram-negative bacilli (including *Pseudomonas* and ESBLs-producing *Enterobacterales*) and synergize with some Gram-positives, but they lack anaerobic activity and carry risks of nephro- and ototoxicity. With limited peritoneal penetration and reduced killing at acidic pH, most authors discourage their routine empiric use in IAIs [52]; however, these antibiotics remain an option to treat patients with Gram-negative resistant infections.

Fosfomycin offers broad activity, excellent tissue penetration, bactericidal action even under anaerobic conditions, and minimal protein binding [53]. The role of intravenous fosfomycin as a part of combination therapy for Gram-negative bacteria needs to be better evaluated in clinical practice, as in vitro data show potential efficacy, and its high tissue penetration may be very attractive for managing patients with IAIs [54].

Even if, for treating ESBL-*Enterobacterales* infections, carbapenems remain the empiric mainstay, ceftolozane–tazobactam and ceftazidime–avibactam (CAZ-AVI) perform well against ESBL-*Enterobacterales*, and can support carbapenem-sparing strategies, if requested [55,56]. Because they lack coverage of anaerobes and certain Gram-positives (e.g., streptococci and methicillin-susceptible *Staphylococcus aureus*), they should be combined with agents active against those organisms.

Observational and some prospective data support CAZ-AVI for BSIs, cIAIs, and complicated urinary tract infections, even with ICU admission up to ~60% [57,58,59,60,61,62,63,64]. Van Duin et al. reported lower 30-day mortality with CAZ-AVI versus colistin-based regimens for CPE infections (9% vs. 32%) and a higher probability of better outcomes at 30 days [57]. Two large Italian series demonstrated the benefit of CAZ-AVI for *Klebsiella pneumoniae* carbapenemase (KPC)-Kp infections: as salvage therapy, it reduced 30-day mortality versus matched non-CAZ-AVI regimens in BSIs (36.5% vs. 55.8%) [58], and in a 577-patient cohort (BSIs and non-bacteremic sites), overall, 30-day mortality was 25% with no difference between monotherapy and combination therapy [59].

These data are confirmed by a recent phase 3 open-label study that evaluated the efficacy and safety of ceftazidime–avibactam in Japanese patients with cIAIs [65].

CAZ-AVI covers most KPC and OXA-48-like CPE and is preferred for OXA-48-like producers [66].

Meropenem–vaborbactam is active against KPC producers. In TANGO II, it yielded higher clinical cure (65.6% vs. 33.3%) and lower mortality (15.6% vs. 33.3%) than best available therapy, though ICU representation was limited [67]; additional observational cohorts in critically ill patients support its use [68,69].

To evaluate the antimicrobial susceptibility of *Enterobacterales* isolated from patients with IAIs in 63 hospitals from the United States (US) medical centers, a retrospective study was recently published. Aztreonam–avibactam, ceftazidime–avibactam, and meropenem–vaborbactam exhibited almost complete activity (99.9% susceptibility) against *Enterobacterales* causing IAIs in US hospitals [70].

Imipenem–relebactam, also active against KPC, outperformed colistin + imipenem in RESTORE-IMI 1 on several endpoints with less nephrotoxicity, albeit with very few cIAI cases enrolled [71]. Against difficult *P. aeruginosa*, Canadian surveillance showed imipenem–relebactam susceptibility of 47.0% (difficult-to-treat resistance) and 71.5% (MDR), second only to ceftolozane–tazobactam and exceeding CAZ-AVI [72]. Meropenem–vaborbactam and imipenem–relebactam generally lack activity against OXA-48-like carbapenemases [73,74], and none of CAZ-AVI, meropenem–vaborbactam, or imipenem–relebactam inhibits metallo-β-lactamase (MBL) producers, though all three remain options for CPE outside the urinary tract where appropriate. Resistance to CAZ-AVI has emerged via numerous KPC variants (>200 described), some conferring cross-resistance to meropenem–vaborbactam and occasionally imipenem–relebactam, complicating treatment optimization and stewardship [75,76,77].

Eravacycline, a fluorocycline, was non-inferior to ertapenem (IGNITE 1) and meropenem (IGNITE 4) for cIAIs (clinical cure ~87–91%) and was associated with very low *Clostridioides difficile* risk [78,79,80]. A meta-analysis of 25 RCTs found eravacycline superior to tigecycline for microbiologic response and comparable in safety and mortality to six other regimens [81]. European Society of Clinical Microbiology and Infectious Diseases Society of America lists eravacycline as an alternative for ESBL- and CPE-related infections (including KPC, MBL, OXA-48), except for primary BSIs and urinary tract infections [82]. Although its large volume of distribution raises concern for bacteremia, a post hoc analysis reported similar microbiologic eradication to comparators in cIAIs with secondary bacteremia [83,84].

Cefiderocol, a siderophore cephalosporin, has broad Gram-negative activity via iron-transport entry, but resistance can arise during therapy. In a phase 3 RCT of carbapenem-resistant infections, cefiderocol achieved comparable clinical and microbiologic cure to the best available therapy, yet mortality was higher in subsets with *Acinetobacter* infections; IAIs were few in both arms [85]. Subsequent uncontrolled studies expanded experience, but more data are needed to refine its role in extensively drug-resistant (XDR) infections [86,87,88,89,90,91].

MBLs require zinc at the active site, hydrolyze carbapenems, and are poorly inhibited by available serine-beta-lactamase inhibitors; they spare aztreonam and show limited activity against monobactams [92]. Because CAZ-AVI does not inhibit MBLs and many MBL producers co-produce other enzymes (ESBL, AmpC, OXA-48), combining CAZ-AVI with aztreonam has been proposed to restore activity [93,94,95].

Aztreonam–avibactam (ATM-AVI) has emerged as an important option against multidrug-resistant Gram-negative bacteria, particularly in infections where therapeutic choices are limited [96]. From a clinical standpoint, ATM-AVI may be especially valuable in situations where resistance mechanisms, such as metallo-beta-lactamase production, limit the activity of other beta-lactams, representing a promising addition to the antimicrobial armamentarium for complicated Gram-negative infections. The Phase 3 ASSEMBLE trial explored the role of ATM-AVI in patients with serious infections caused by MBL-producing multidrug-resistant organisms. These infections, ranging from IAIs and urinary tract infections to pneumonia and BSIs, represent some of the most challenging scenarios in contemporary clinical practice due to limited therapeutic options [96]. In this multicenter, randomized, assessor-blinded study, patients received either ATM-AVI (with metronidazole for intra-abdominal cases) or best available therapy. Although the overall sample size was small, the majority of participants were infected with *K. pneumoniae*, and several MBL variants were identified, particularly NDM and VIM types. Clinical outcomes favored ATM-AVI, with nearly half of patients in this arm achieving a cure compared with none in the control group. Mortality was also lower among ATM-AVI recipients, and microbiological results aligned closely with clinical responses. Importantly, the regimen was generally well tolerated, with no serious drug-related safety concerns emerging. Despite the limited number of patients, these findings add to the growing body of evidence that ATM-AVI could become an important treatment option for MBL-producing Gram-negative infections, an area where few effective alternatives exist. Larger studies will be needed to confirm these results and define their role across different infection types.

Among non-fermenters (*Pseudomonas aeruginosa*, *Stenotrophomonas maltophilia*, *Acinetobacter baumannii*), alarming hospital resistance rates are reported worldwide, driven by intrinsic and acquired mechanisms.

For Gram-positive pathogens, the mortality impact of *Enterococcus* spp. in IAIs remains uncertain [97]. While enterococci are clearly implicated in breakthrough or superinfections in high-risk hosts, their role in low-risk IAI is debated [98]. Low intrinsic virulence may be offset by synergy with organisms like *E. coli* and anaerobes [98,99]. Observational studies link enterococcal isolation to poorer outcomes, yet RCTs have not shown survival benefit from routine anti-enterococcal coverage, suggesting *Enterococcus* may be more of a prognostic marker than a principal driver of disease [100,101,102]. A meta-analysis by Zhang et al. found no improvement in treatment success, mortality, or adverse events with enterococcal-active empiric therapy versus control regimens [29]. Risk factors for enterococcal IAI include malignancy, corticosteroids, prior antibiotics, ICU admission, indwelling catheters, and nosocomial acquisition (odds ratio 2.81) [29]. In a multicenter study with ~65% CA-IAIs, outcomes at 30 days did not differ between patients with *E. faecalis* treated empirically with piperacillin–tazobactam versus ertapenem (which lacks reliable enterococcal coverage) [103].

A pragmatic takeaway from the literature is to consider empiric enterococcal coverage in: (1) immunocompromised hosts or hospital-acquired/post-operative cIAIs; (2) prior cephalosporin/broad-spectrum exposure selecting for enterococci; and (3) high risk of endocarditis (valvular disease, intravascular prostheses) [104]. Most *Enterococcus faecalis*, including some vancomycin-resistant *Enterococcus* (VRE), remain ampicillin-susceptible, whereas *E. faecium* is often ampicillin- and increasingly vancomycin-resistant [105,106]. BSIs are the major clinical concern. A recent meta-analysis showed higher mortality with VRE *E. faecium* BSIs versus vancomycin-susceptible strains [107]. For VanA-type VRE *E. faecium*, linezolid or high-dose daptomycin is preferred; for VanB-type, teicoplanin is an option [105,106].

## 4. How

Antibiotic dosing should be tailored to the pharmacokinetic (PK) and pharmacodynamic (PD) properties of the drug class and agent, as well as to the patient’s pathophysiology.

PD describes how exposure relates to antibacterial effects. The minimum inhibitory concentration (MIC) is the core in vitro metric of activity. For time-dependent agents (e.g., β-lactams), drug levels at the infection site should remain above the MIC for at least ~40% of the dosing interval—and ideally much longer, up to 400% (e.g., 100% fT > 4 × MIC). For concentration-dependent agents (e.g., aminoglycosides), efficacy correlates with high peaks, targeting Cmax/MIC > 8–10 [108]. PK determines the time course of drug concentrations through absorption, distribution, metabolism, and elimination; inadequate exposure at the target site risks clinical failure and fosters resistance, especially with borderline MICs [108]. Tissue penetration is critical, typically greater for lipophilic than hydrophilic drugs, though disease factors can modify distribution [108]. In severe IAIs, higher doses may be required to achieve adequate levels of ceftazidime, meropenem, and imipenem [109,110,111,112]. In a 2020 prospective observational study of critically ill surgical patients started on β-lactams, high dosing achieved 100% serum fT > 4 × MIC within 24 h in 78% of severe IAI cases. Optimal β-lactam dosing should therefore consider both tissue penetration and local susceptibility patterns [113]. Because sepsis alters physiology, PK can vary widely; the patient’s pathophysiologic and immune status must guide dosing decisions [114]. The “dilution effect” or “third spacing” particularly affects hydrophilic agents (β-lactams, aminoglycosides, glycopeptides) that distribute mainly in extracellular fluid. Without an adequate loading dose, peritoneal (and other) sites underexposure can lead to failure and resistance.

Accordingly, in sepsis or septic shock, initial loading doses of β-lactams or glycopeptides should be ~1.5× higher than in stable patients to rapidly attain therapeutic levels [114]. Subsequent maintenance dosing must be adjusted to renal function and reassessed daily, since dynamic physiologic changes alter drug clearance. Reduce doses in renal impairment and increase when augmented renal clearance is present (creatinine clearance > 130 mL/min) [114,115]. Because serum creatinine is unreliable in the critically ill, measure urinary creatinine clearance to better gauge renal function [116].

Dosing strategies should reflect whether activity is time- or concentration-dependent. β-lactams perform best when troughs (Cmin) stay above the MIC (Cmin > MIC) [109]. More frequent dosing, prolonged infusions, or continuous infusions can improve target attainment; with the same total daily dose, extended/continuous infusions raise the chance of maintaining Cmin > MIC [114]. Large RCTs comparing continuous versus intermittent infusion of piperacillin/tazobactam in cIAIs, and of piperacillin/tazobactam, ticarcillin/clavulanate, or meropenem in severe sepsis, did not show overall clinical benefit for continuous infusion [114]. However, these results should not be overgeneralized to patients with high severity and/or pathogens with borderline high MICs—groups most likely to benefit per PK/PD principles; several retrospective studies support this nuance [117,118,119]. Thus, prolonged or continuous β-lactam infusions are reasonable for severely ill abdominal sepsis patients, especially where MDR prevalence is high.

A recent multicenter RCT found no superiority of continuous over intermittent meropenem infusion in septic ICU patients for 28-day mortality or emergence of Pan-drug resistant [PDR]/XDR organisms [120], though potential biases (late randomization, baseline severity, sample size) limit firm conclusions [121]. Overall, extended/continuous β-lactam infusion remains a valuable strategy in critically ill patients with abdominal sepsis. By contrast, concentration-dependent drugs should maximize Cmax/MIC (>8–10), favoring once-daily “pulse” dosing [114].

For aminoglycosides specifically, once-daily dosing also reduces nephrotoxicity versus multiple daily dosing, likely by saturating renal cortical uptake mechanisms and limiting accumulation [122].

## 5. How Long

Evidence strongly supports the safety of short-duration antibiotic courses in patients with cIAIs, provided adequate source control has been achieved [123].

The STOP-IT trial by Sawyer et al. [124] demonstrated that a fixed four-day regimen produced outcomes comparable to those of extended therapy continued until the resolution of physiological abnormalities.

In acute appendicitis, treatment length may be further reduced. A recent open-label, non-inferiority trial in patients ≥8 years with complex appendicitis showed that two days of postoperative intravenous antibiotics was not inferior to five days regarding infectious complications and 90-day mortality, using a non-inferiority margin of 7.5% [125].

Short antibiotic regimens have also proven effective in postoperative peritonitis. A multicenter RCT conducted in 21 French ICUs (2011–2015) compared 8-day versus 15-day antibiotic courses in critically ill patients. The shorter regimen led to more antibiotic-free days (*p* < 0.0001) while showing no difference in 45-day mortality, ICU/hospital stay, multidrug-resistant infections, or reoperation rates. These results confirm that extending therapy to 15 days does not provide additional clinical benefit [126]. A retrospective cohort study of 42 surgical ICU patients with BSIs secondary to IAIs also showed that stopping antibiotics within seven days of achieving adequate source control did not increase recurrence risk [127].

Despite these findings, variations in practice remain, especially in critically ill patients, due to the lack of universally applicable data. Many such patients still receive unnecessarily prolonged therapy. For individuals with ongoing infection signs, treatment duration should be individualized. Monitoring inflammatory biomarkers such as C-reactive protein and procalcitonin (PCT) may assist in decisions to continue, narrow, or discontinue therapy. Patients who still show systemic illness or infection signs after seven days of antibiotics should undergo repeat diagnostic evaluation to rule out uncontrolled sources or treatment failure [128]. Subgroup analysis of the STOP-IT trial identified risk factors for treatment failure, including corticosteroid use, APACHE II scores ≥ 5, healthcare-associated infections, and colonic sources of infection [129]. Nonetheless, even in these higher-risk patients, extended antibiotic courses did not reduce failure rates, indicating no advantage from prolonged therapy.

PCT has recently emerged as a valuable tool for tailoring antibiotic duration in critically ill patients [130,131,132,133,134]. Several studies suggest that PCT-guided strategies safely shorten therapy in cIAIs [135,136,137].

## 6. Conclusions

IAIs represent a complex clinical spectrum requiring timely diagnosis, surgical source control, and judicious antibiotic use. Evidence increasingly supports tailored, shorter courses of therapy once adequate source control is achieved, thereby minimizing toxicity and resistance selection without compromising outcomes. Empiric therapy must balance urgency with precision, taking into account local epidemiology, patient-specific risk factors, and the rising prevalence of MDR organisms. Advances in antibiotic agents, optimized PK/PD strategies, and stewardship-driven de-escalation provide opportunities to improve efficacy while preserving future antibiotic utility. Ultimately, the challenge lies in integrating surgical, microbiological, and pharmacological principles to ensure effective, individualized, and responsible management of IAIs in an era of escalating antimicrobial resistance.

## Data Availability

No new data were created or analyzed in this study. Data sharing is not applicable to this article.

## Abbreviations

The following abbreviations are used in this manuscript:

AGNB	Aerobic Gram-negative bacteria
AMR	Antimicrobial resistance
ATM-AVI	Aztreonam–avibactam
BSIs	Bloodstream infections
CAZ-AVI	Ceftazidime–avibactam
cIAI	Complicated intra-abdominal infection
Cmax	Peak plasma concentration
CPE	Carbapenemase-producing *Enterobacterales*
CT	Computed tomography
ESBL	Extended-spectrum beta-lactamase
fT	Dosing interval
HAI	Healthcare associated infection
IAI	Intra-abdominal infection
ICU	Intensive care unit
KPC	*Klebsiella pneumoniae* carbapenemase
MBL	Metallo-beta-lactamase
MDR	Multi-drug resistant
MIC	Minimum inhibitory concentration
PCT	Procalcitonin
PD	Pharmacodynamic
PK	Pharmacokinetic
RCT	Randomized controlled trial
VRE	Vancomycin-resistant *Enterococcus*
XDR	Extensively drug resistant
